# Allele frequencies of 15 STR loci in Bosnian and Herzegovinian population

**DOI:** 10.3325/cmj.2017.58.250

**Published:** 2017-06

**Authors:** Amela Pilav, Naris Pojskić, Anesa Ahatović, Mirela Džehverović, Jasmina Čakar, Damir Marjanović

**Affiliations:** 1 University of Sarajevo-Institute for Genetic Engineering and Biotechnology, Sarajevo, Bosnia and Herzegovina; 2 International Burch University, Sarajevo, Bosnia and Herzegovina; 3 Institute for Anthropological Research, Zagreb, Croatia

## Abstract

**Aim:**

To determine newest the most accurate allele frequencies for 15 short tandem repeat (STR) loci in the Bosnian and Herzegovinian population, calculate statistical parameters, and compare them with the relevant data for seven neighboring populations.

**Methods:**

Genomic DNA was obtained from buccal swabs of 1000 unrelated individuals from all regions of Bosnia and Herzegovina. Genotyping was performed using PowerPlex^®^ 16 System to obtain allele frequencies for 15 polymorphic STR loci including D3S1358, TH01, D21S11, D18S51, Penta E, D5S818, D13S317, D7S820, D16S539, CSF1PO, Penta D, vWA, D8S1179, TPOX, and FGA. The calculated allele frequencies were also compared with the data from neighboring populations.

**Results:**

The highest detected value of polymorphism information content (PIC) was detected at the PentaE locus, whereas the lowest value was detected at the TPOX locus. The power of discrimination (PD) values had similar distribution, with Penta E showing the highest PD of 0.9788. While D18S51 had the highest value of power of exclusion (PE), the lowest PE value was detected at the TPOX locus.

**Conclusion:**

Upon comparison of Bosnian and Herzegovinian population data with those of seven neighboring populations, the highest allele frequency differentiation was noticed between Bosnian and Herzegovinian and Turkish population at 5 loci, the most informative of which was Penta E. The neighbor-joining dendrogram constructed on the basis of genetic distance showed grouping of Slovenian, Austrian, Hungarian, and Croatian populations. Bosnian and Herzegovinian population was between the mentioned cluster and Serbian population. To determine more accurate distribution of allelic frequencies and forensic parameters, our study included 1000 unrelated individuals from all regions of Bosnia and Herzegovina, and our findings demonstrated the applicability of these markers in both forensics and future population genetic studies.

Short Tandem Repeats (STRs) are common markers in population biodiversity research, paternity testing, and forensic analysis of biological evidence. Reliability of STR amplification provides a high level of individualization that is crucial for population genetic studies. To obtain precise and reliable results of analysis, it is necessary to use population data obtained from a sufficient number of the samples (1). Currently available official allele frequencies for Bosnian and Herzegovinian population at 15 STR loci addressed in this study were published 10 years ago and obtained from only 100 unrelated individuals, which was acceptable at the time (2).

The latest recommendations regarding the official publication and forensic usage of STR population data highlighted the need for increasing the size of population sample for its calculation. Therefore, the main aim of this study was to determine latest and more accurate allele frequencies and forensic statistical parameters for 15 most used STR loci in the Bosnian and Herzegovinian population and compare them with the relevant data for other neighboring populations.

## MATERIAL AND METHODS

### Material

Biological buccal swab samples were collected from 1000 unrelated individuals from all regions of Bosnia and Herzegovina. Samples were randomly collected from routine casework performed during the period of 2006-2016 at the Institute for genetic engineering and biotechnology, University of Sarajevo. Only unrelated adults over 18 years of age were included in this study. Informed consent for the use of collected biological material and data was obtained from all subjects.

### DNA analysis

DNA extraction was performed using the Qiagen DNeasyTM Tissue Kit (3). DNA concentration was determined using Quantifiler® Duo DNA Quantification Kit in 7500 RealTime PCR System (Applied Biosystems, Foster City, CA, USA). Genotypes at 15 autosomal STR loci (D3S1358, TH01, D21S11, D18S51, Penta E, D5S818, D13S317, D7S820, D16S539, CSF1PO, Penta D, vWA, D8S1179, TPOX, FGA) and amelogenin were obtained using PowerPlex® 16 System (Promega, Madison, WI, USA) (4). PCR was performed according to the recommendations in 25-µL reactions. Amplification was carried out in a GeneAmp PCR System 9700 (Applied Biosystems). Fragment analysis was performed in ABI PRISM® 310 Genetic Analyzer (Applied Biosystems). A mix of 11.5 µL of formamide, 0.5 µL of Internal Lane Standard 600 (Promega) and 1 µL of PCR product was run under recommended conditions. GeneMapper® ID Software version 3.2 (Applied Biosystems) was used for raw data analysis.

### Statistical analysis

Allele frequencies, matching probability (MP), power of discrimination (PD), power of exclusion (PE), and typical paternity index (TPI) were calculated within Microsoft Excel workbook template – PowerStats (5). Powermarker version 3.25 was used for estimation of allele number (AN) (6), deviation from Hardy–Weinberg equilibrium (7), polymorphism information content (8), and observed and expected heterozygosity (9). Exact test of population differentiation (10) was calculated within Arlequin version 3.5.1.2 (11). After Bonferroni’s correction, statistically significant deviation from Hardy–Weinberg equilibrium was considered as *P* < 0.01 and *P* < 0.001 for the population differentiation test. The number of effective alleles (AE) was estimated as 1/ ∑ pi2, where p denotes the allele frequency for a particular locus. Ratio of effective and detected numbers of alleles and its statistical significance was calculated as suggested by Pojskic et Kalamujic (12) with Alleles Ratio, a Microsoft Excel workbook template (13). A Z-score of *P* < 0.01 was considered statistically significant. In order to estimate genetic distance among populations, we have implemented Dsw method proposed by Shriver et al (14). The neighbor-joining dendrogram was constructed based on genetic distance results (15). These calculations were performed using POPTREE software (16).

## RESULTS

Allele frequencies and statistical parameters including heterozygosity (observed and expected), results of exact test, PD, and PE for the 15 STR markers were calculated (Tables 1 and 2). No statistically significant deviation from Hardy-Weinberg equilibrium was found at analyzed loci (*P* > 0.05 for all), except at the D8S1179 locus. However, after applying the Bonferroni’s correction, no statistical significance was revealed either (*P* = 0.015). Excess of heterozygosity was detected for D3S1358, D21S11, D18S51, D16S539, vWA, and TPOX loci (Table 2). A total of 160 alleles were detected, with 32 of those qualifying as rare alleles (frequency <0.005). The highest number of alleles was detected at the Penta E locus (allele 18) and the lowest at the TH01locus (allele 7) (Table 1). The highest number of effective alleles was estimated for the PentaE (9.47) and lowest for the TPOX locus (2.55). The highest ratio between the number of effective and observed alleles (A_E_/A_N_) was detected for the TH01 (0.652) and lowest for the TPOX (0.319) locus (Table 2). However, D21S11 (Z = 3.420, *P* = 0.001), D18S51 (Z = 3.019, *P* = 0.003), Penta E (Z = 3.344, *P* = 0.001), PentaD (Z = 2.621, *P* = 0.009), D8S1179 (Z = 2.616, *P* = 0.009), TPOX (Z = 2.874, *P* = 0.004) and FGA (Z = 3.699, *P* < 0.001) loci showed statistically significant ratio effective and detected number of alleles (*P* < 0.01) indicating a sharp-cut departure of effective number of alleles from the detected number of alleles.

**Table 1:** Allele frequencies for 15 short tandem repeat (STR) loci profiled with PowerPlex^®^ 16 System in a Bosnian and Herzegovinian population (N = 1000)*

	STR loci														
Allele	D3S1358	TH01	D21S11	D18S51	Penta_E	D5S818	D13S317	D7S820	D16S539	CSF1PO	Penta_D	vWA	D8S1179	TPOX	FGA
5	-	0.002	-	-	0.070	-	-	-	-	-	-	-	-	-	-
6	-	0.258	-	-	0.001	-	-	-	-	-	-	-	-	0.001	-
7	-	0.125	-	-	0.146	0.003	-	0.013	-	-	0.002	-	-	0.004	-
8	-	0.105	-	-	0.012	-	0.129	0.165	0.018	0.002	0.012	-	0.014	0.561	-
9	-	0.219	-	0.002	0.014	0.035	0.090	0.162	0.097	0.034	0.226	-	0.017	0.090	-
9.3	-	0.280	-	-	-	-	-	-	-	-	-	-	-	-	-
10	-	0.012	-	0.011	0.131	0.073	0.050	0.278	0.053	0.287	0.104	-	0.069	0.063	-
11	-	-	-	0.019	0.100	0.318	0.355	0.215	0.296	0.295	0.187	-	0.062	0.254	-
12	0.001	-	-	0.110	0.162	0.381	0.268	0.138	0.309	0.320	0.182	0.001	0.167	0.028	-
13	0.002	-	-	0.120	0.124	0.175	0.083	0.024	0.200	0.050	0.199	0.005	0.315	0.001	-
14	0.096	-	-	0.190	0.054	0.015	0.026	0.006	0.026	0.011	0.061	0.115	0.245	-	-
15	0.275	-	-	0.134	0.058	0.002	0.001	-	0.003	0.003	0.018	0.117	0.092	-	-
16	0.246	-	-	0.169	0.039	-	-	-	-	-	0.007	0.217	0.022	-	0.001
17	0.210	-	-	0.095	0.049	-	-	-	-	-	0.004	0.260	0.001	-	0.001
18	0.159	-	-	0.063	0.025	-	-	-	-	-	-	0.197	-	-	0.009
19	0.014	-	-	0.037	0.011	-	-	-	-	-	-	0.076	-	-	0.089
20	0.001	-	-	0.024	0.006	-	-	-	-	-	-	0.015	-	-	0.121
21	-	-	-	0.023	0.003	-	-	-	-	-	-	-	-	-	0.179
21.2	-	-	-	-	-	-	-	-	-	-	-	-	-	-	0.006
22	-	-	-	0.005	0.001	-	-	-	-	-	-	0.001	-	-	0.212
22.2	-	-	-	-	-	-	-	-	-	-	-	-	-	-	0.008
23	-	-	-	0.001	-	-	-	-	-	-	-	-	-	-	0.135
23.2	-	-	-	-	-	-	-	-	-	-	-	-	-	-	0.006
24	-	-	-	-	-	-	-	-	-	-	-	-	-	-	0.112
24.2	-	-	-	-	-	-	-	-	-	-	-	-	-	-	0.001
25	-	-	0.001	-	-	-	-	-	-	-	-	-	-	-	0.084
26	-	-	0.003	-	-	-	-	-	-	-	-	-	-	-	0.031
27	-	-	0.030	-	-	-	-	-	-	-	-	-	-	-	0.008
28	-	-	0.156	-	-	-	-	-	-	-	-	-	-	-	0.001
29	-	-	0.218	-	-	-	-	-	-	-	-	-	-	-	-
29.2	-	-	0.003	-	-	-	-	-	-	-	-	-	-	-	-
30	-	-	0.227	-	-	-	-	-	-	-	-	-	-	-	-
30.2	-	-	0.038	-	-	-	-	-	-	-	-	-	-	-	-
31	-	-	0.061	-	-	-	-	-	-	-	-	-	-	-	-
31.2	-	-	0.102	-	-	-	-	-	-	-	-	-	-	-	-
32	-	-	0.013	-	-	-	-	-	-	-	-	-	-	-	-
32.2	-	-	0.099	-	-	-	-	-	-	-	-	-	-	-	-
33.2	-	-	0.045	-	-	-	-	-	-	-	-	-	-	-	-
34.2	-	-	0.006	-	-	-	-	-	-	-	-	-	-	-	-
35.2	-	-	0.001	-	-	-	-	-	-	-	-	-	-	-	-

**Table 2:** Statistical data for 15 short tandem repeat (STR) loci profiled with PowerPlex^®^ 16 System in a Bosnian and Herzegovinian population (N = 1000)*

Statistical parameter	STR loci														
	D3S1358	TH01	D21S11	D18S51	Penta_E	D5S818	D13S317	D7S820	D16S539	CSF1PO	Penta_D	vWA	D8S1179	TPOX	FGA
H_obs_	0.807	0.764	0.855	0.885	0.877	0.704	0.766	0.786	0.771	0.709	0.825	0.824	0.781	0.613	0.856
H_exp_	0.786	0.781	0.848	0.875	0.894	0.717	0.768	0.803	0.764	0.725	0.827	0.814	0.796	0.608	0.862
P	0.886	0.836	0.194	0.666	0.293	0.820	0.570	0.606	0.451	0.082	0.731	0.418	0.015	0.956	0.817
A_N_	9	7	15	15	18	8	8	8	8	8	11	10	10	8	17
A_E_	4.67	4.56	6.59	8.01	9.47	3.53	4.31	5.09	4.24	3.63	5.76	5.39	4.90	2.55	7.24
A_E_/A_N_	0.519	0.651	0.439	0.534	0.526	0.441	0.538	0.636	0.530	0.454	0.524	0.539	0.490	0.319	0.426
PD	0.918	0.917	0.957	0.971	0.979	0.873	0.913	0.933	0.905	0.876	0.946	0.938	0.929	0.797	0.965
PE	0.612	0.534	0.705	0.765	0.749	0.435	0.538	0.573	0.546	0.442	0.646	0.644	0.564	0.307	0.707
PIC	0.752	0.746	0.831	0.863	0.885	0.669	0.735	0.775	0.727	0.674	0.803	0.789	0.768	0.558	0.847
MP	0.082	0.083	0.043	0.029	0.021	0.127	0.087	0.067	0.095	0.124	0.055	0.062	0.071	0.203	0.035
TPI	2.591	2.119	3.448	4.348	4.065	1.689	2.137	2.336	2.183	1.718	2.857	2.841	2.283	1.292	3.472

*Abbreviations: H_obs_ - observed heterozygosity; H_exp_ - expected heterozygosity; P - deviation from Hardy–Weinberg equilibrium; A_N_ – number of detected alleles; A_E_ – effective number of alleles; A_E_/A_N_ – ratio between the number of effective and detected alleles; PD – power of discrimination; PE – power of exclusion; PIC – polymorphism information content; MP – matching probability, and TPI – typical paternity index.

The highest value of polymorphism information content (PIC) was found for the Penta E, and the lowest for the TPOX locus. The same results were obtained for PD, while the highest PE value was detected for D18S51 (Table 2). The lowest matching probability was observed for the Penta E locus, whereas the highest TPI was calculated for the D18S51 (Table 2). Statistically significant differences were found in allele frequencies between the Bosnian and Herzegovinian population and data available of seven neighboring populations (Table 3). The largest differences were found between the Bosnian and Herzegovinian population and Turkish, Croatian, Austrian, and Italian population (Table 3).

**Table 3:** Comparison of allele frequencies on 15 short tandem repeat (STR) loci between Bosnian and Herzegovinian population and previously published population data*

	Population (*P* value ± standard error)						
Locus	Croatia	Slovenia	Serbia	Turkey	Hungary	Austria	Italy
D3S1358	0.004 ± 0.002	0.313 ± 0.067	0.657 ± 0.055	0.220 ± 0.073	0.181 ± 0.028	0.529 ± 0.034	0.485 ± 0.094
TH01	0.002 ± 0.002	0.151 ± 0.055	0.021 ± 0.008	0.00000 ± 0.0000	0.013 ± 0.004	0.00000 ± 0.0000	0.079 ± 0.035
D21S11	0.988 ± 0.006	0.316 ± 0.067	0.220 ± 0.054	0.056 ± 0.018	0.141 ± 0.030	0.442 ± 0.063	0.230 ± 0.098
D18S51	0.011 ± 0.004	0.193 ± 0.031	0.895 ± 0.033	0.013 ± 0.007	0.230 ± 0.056	0.003 ± 0.002	0.001 ± 0.001
Penta_E	-	0.976 ± 0.007	-	-	0.087 ± 0.025	-	-
D5S818	-	0.240 ± 0.035	0.218 ± 0.048	0.138 ± 0.032	0.001 ± 0.001	-	-
D13S317	-	0.760 ± 0.037	0.273 ± 0.071	0.002 ± 0.002	0.072 ± 0.032	-	-
D7S820	-	0.080 ± 0.024	0.031 ± 0.011	0.592 ± 0.037	0.009 ± 0.005	-	-
D16S539	0.8240 ± 0.028	0.917 ± 0.023	0.450 ± 0.076	0.00000 ± 0.0000	0.587 ± 0.040	0.001 ± 0.001	0.00000 ± 0.0000
CSF1PO	-	0.298 ± 0.057	0.435 ± 0.056	0.336 ± 0.052	0.758 ± 0.051	-	-
Penta_D	-	0.377 ± 0.058	-	-	0.016 ± 0.008	-	-
vWA	0.011 ± 0.006	0.002 ± 0.002	0.766 ± 0.058	0.176 ± 0.043	0.078 ± 0.024	0.126 ± 0.062	0.105 ± 0.032
D8S1179	0.00000 ± 0.0000	0.583 ± 0.032	0.201 ± 0.057	0.00000 ± 0.0000	0.013 ± 0.005	0.005 ± 0.004	0.006 ± 0.002
TPOX	-	0.166 ± 0.023	0.459 ± 0.058	0.853 ± 0.024	0.146 ± 0.044	-	-
FGA	0.005 ± 0.003	0.493 ± 0.050	0.354 ± 0.060	0.001 ± 0.001	0.160 ± 0.0356	0.018 ± 0.018	0.496 ± 0.124

*P-value of the exact test of population differentiation. Considered level of statistical significance: *P* < 0.005 – D3S1358, TH01, D21S11, D18S51, D16S539, vWA, D8S1179, FGA; *P* < 0.008 – D5S818, D13S317, D7S820, CSF1PO, TPOX; *P* < 0.015 – Penta E, Penta D.

**Figure 1:**
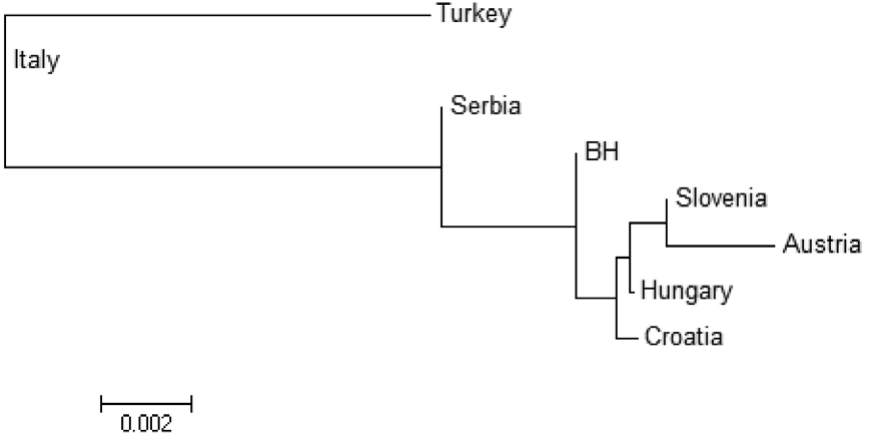
The neighbor-joining dendrogram showing the relationship between Bosnian and Herzegovinian population and seven other populations based on results of genetic distance analysis.

The neighbor-joining dendrogram based on result of genetic distance analysis showed the relationship between the Bosnian and Herzegovinian population and neighboring populations (17-23). It showed that the Bosnian and Herzegovinian population had the greatest genetic distance from Turkish (0.220) and the smallest genetic distance from the Slovenian (0.001) and Hungarian populations (0.001) (Figure 1 and Table 4).

**Table 4:** Genetic distance analysis between the population in Bosnia and Herzegovina and seven other populations

	Bosnia and Herzegovina	Croatia	Slovenia	Serbia	Turkey	Austria	Italy	Hungary
Bosnia and Herzegovina	-	0.001	0.001	0	0.022	0.003	0.007	0.001
Croatia	-	-	0	0.003	0.024	0.004	0.007	0.001
Slovenia	-	-	-	0.002	0.021	0.001	0.007	-0.001
Serbia	-	-	-	-	0.014	0.005	0.006	0.002
Turkey	-	-	-	-	-	0.03	0.005	0.023
Austria	-	-	-	-	-	-	0.013	0.004
Italy	-	-	-	-	-	-	-	0.008
Hungary	-	-	-	-	-	-	-	-

## DISCUSSION

Our results were concordant with the findings of the previous study conducted in Bosnian and Herzegovinian population (2). This was also demonstrated in previous studies for other populations (17-23). Our results showed that locus D18S51 had the highest PE and TPI values. The most discriminating STR loci in Bosnian and Herzegovinian population were Penta E and D18S51. Therefore, it should be desirable to include these two loci in paternity testing and forensic analysis of biological evidence.

Allele frequencies for 15 STR loci in the studied Bosnian and Herzegovinian population did not differ significantly from those found in populations of Slovenia (17), Serbia (18), and Hungary (19). In other words, according to 15 analyzed STR loci here, the Bosnian and Herzegovinian population is genetically more similar to Slovenian, Serbian, and Hungarian populations than to the remaining four populations. Also, no significant differences were observed in previous population studies (17,18) when compared with earlier Bosnian and Herzegovinian data (2). Statistically significant differences between Bosnian and Herzegovinian and Turkish populations were found at five loci (TH01, D13S317, D16S539, D8S1179 and FGA) (19) and among Bosnian and Herzegovinian and Croatian population at 4 loci (D3S1358, TH01, D8S1179 and FGA) (20). A deviation of allele frequencies in Bosnian and Herzegovinian population from those in Croatian population was observed in the previous study (2) at the locus D8S1179. Also, Bosnian and Herzegovinian allele frequencies differed from Austrian (21) at 4 loci (TH01, D18S51, D16S539 and D8S1179), whereas deviation of Bosnian and Herzegovinian allele frequencies distribution from Italian (22) was observed only for two loci (D18S51 and D16S539).

The neighbor-joining dendrogram showed the relationship among 8 populations on the basis of result of genetic distance analysis. Bosnian and Herzegovinian population has the highest genetic distance from the Turkish population and the lowest from the Serbian population. The neighbor-joining dendrogram constructed on the basis of genetic distance showed grouping of Slovenian, Austrian, Hungarian, and Croatian populations. Bosnian and Herzegovinian population is placed between the mentioned cluster and the Serbian population. The results of genetic distance analysis are in concordance with conclusions about similarity among neighboring populations based on of STR profiles (24).

Although a study that was based on 100-150 respondents was sufficient at the time (25) and considered adequate for determining parameters for the given population, the latest recommendations regarding official publication and forensic usage of STR population data highlight the need of increasing the size of a studied population sample. The previous study of the population of Bosnia and Herzegovina analyzed fewer than 150 individuals and DNA typing included STR markers (2). Our findings on the distribution of allelic frequencies and forensic parameters obtained in 1000 unrelated individuals from all regions of Bosnia and Herzegovina demonstrate the applicability of these markers in both forensics and future population genetic studies.
